# Clear Cell Carcinoma of the Vagina in an 11-Year-Old Girl: A Case Report on Clinical, Imaging, and Immunophenotypic Features

**DOI:** 10.7759/cureus.98068

**Published:** 2025-11-29

**Authors:** Amaranto Suárez, Alejandra Beatriz Tijo López, Yuli Natalia Otero Pabon, Oscar Mauricio Forero Cuellar, Adriana Bryon Gallego

**Affiliations:** 1 Pediatric Oncology, Instituto Nacional de Cancerología, Bogotá, COL; 2 Radiation Oncology, Instituto Nacional de Cancerología, Bogotá, COL; 3 Diagnostic Imaging, Instituto Nacional de Cancerología, Bogotá, COL; 4 Oncologic Pathology, Instituto Nacional de Cancerología, Bogotá, COL

**Keywords:** adenocarcinoma of the vagina, adolescent girl, clear cell adenocarcinoma, pediatrics chemotherapy, radiotherapy (rt)

## Abstract

Vaginal clear cell carcinoma (CCC) is a rare malignant tumor in the pediatric population and may occur even in the absence of in utero exposure to diethylstilbestrol (DES). We report the case of an 11-year-old girl without DES exposure who presented with persistent foul-smelling vaginal discharge, abdominal pain, and weight loss. Imaging revealed a large vaginal mass with regional lymph node involvement. Histopathology confirmed CCC with Müllerian immunophenotype. The disease was classified as advanced and unresectable, and the patient was treated with concurrent cisplatin-based chemoradiation followed by high-dose-rate interstitial brachytherapy. She achieved significant tumor reduction and remains clinically disease-free to date. This case underscores the diagnostic complexity of pediatric vaginal CCC and supports chemoradiation with brachytherapy as an effective strategy for unresectable disease.

## Introduction

Gynecologic tumors are uncommon in the pediatric population, accounting for less than 5% of all childhood malignancies according to most series [1,2]. Among them, clear cell carcinoma (CCC) of the vagina represents an exceptional entity, described predominantly in postmenopausal women and not associated with human papillomavirus (HPV) infection [3].

Recent literature suggests a causal association between in utero exposure to diethylstilbestrol (DES) and the development of this carcinoma, attributed to molecular alterations that promote carcinogenesis [4]. These include overexpression of proto-oncogenes (c-jun, c-fos, c-myc), anti-apoptotic genes (bcl-2, bcl-x), and growth factors (EGF, TGF-α), as well as activation of estrogen-responsive genes such as lactoferrin [5].

Clinically, vaginal CCC typically occurs in girls with a mean age of 15 years and presents with abdominal pain, a pelvic or abdominal mass, bloody vaginal discharge, or, in some cases, a protruding vaginal mass. The primary differential diagnoses in pediatric patients include rhabdomyosarcoma and germ cell tumors, which require complementary studies, including tumor markers and histopathological analysis, including immunohistochemistry [2].

Treatment is not yet standardized and depends on the clinical stage at diagnosis. In general, surgery is advisable as initial management for resectable tumors, with or without adjuvant radiotherapy. In other cases, image-guided external beam radiotherapy (EBRT) combined with radiosensitizing chemotherapy, followed by brachytherapy, is employed [6].

We present this case to highlight the diagnostic complexity and therapeutic challenges of vaginal CCC in the pediatric population, emphasizing the importance of a multidisciplinary approach and individualized treatment strategies in this exceptionally rare malignancy, where evidence is limited to case reports and small series.

## Case presentation

An 11-year-old girl with no significant medical history arrived at the Instituto Nacional de Cancerología (Colombia) with a diagnosis of vaginal clear cell adenocarcinoma. Over the course of seven months, she developed a foul-smelling brown vaginal discharge that later became hemorrhagic. The patient reported mild to moderate colicky lower abdominal pain, fatigue, asthenia, intermittent fever, and weight loss of approximately 4 kg. The initial clinical impression was vaginitis, and further evaluation excluded sexual abuse.

Persistent symptoms prompted an MRI of the abdomen and pelvis, which revealed a solid, heterogeneous mass with irregular margins in the vaginal canal. A biopsy confirmed the diagnosis of clear cell carcinoma. Immunohistochemistry revealed diffuse positivity for CK7 and PAX8, focal positivity for Napsin A, and negativity for CK20, estrogen receptors, and WT1. The patient had no history of in utero DES exposure.

Upon admission, the patient had heavy, foul-smelling vaginal discharge requiring multiple sanitary pads during the day and diapers at night. Physical examination revealed generalized mucocutaneous pallor, tenderness on deep palpation of the hypogastrium and left iliac fossa, and a palpable pelvic mass approximately 1 cm from the vaginal introitus. The rest of the examination was unremarkable.

Laboratory studies revealed microcytic, hypochromic anemia (Hb: 9.3 g/dL) and mild thrombocytosis (697,000/mm³); renal and hepatic function tests were normal (Table 1).

**Table 1:** Laboratory findings of the patient with corresponding reference ranges Reference ranges are based on the standards of our institution's laboratory.

Parameter	Result	Reference range	Interpretation
White blood cells (WBC)	7890 cells /uL	4000 - 10000	Normal
Hemoglobin (Hb)	9,3 g/dl	11 - 16	Low
Mean corpuscular volume (MCV)	70,3 fL	80 - 100	Low
Platelets	697000 cells /uL	150000 – 450000	Mild thrombocytosis
Serum creatinine	0,42 mg/dL	0,66 – 1,25	Normal
Blood urea nitrogen	4,95 mg/dL	7 -18	Normal
Aspartate aminotransferase (AST)	11,6 U/L	30 – 65	Normal
Alanine aminotransferase (ALT)	16,8 U/L	15 - 37	Normal

**Figure 1:**
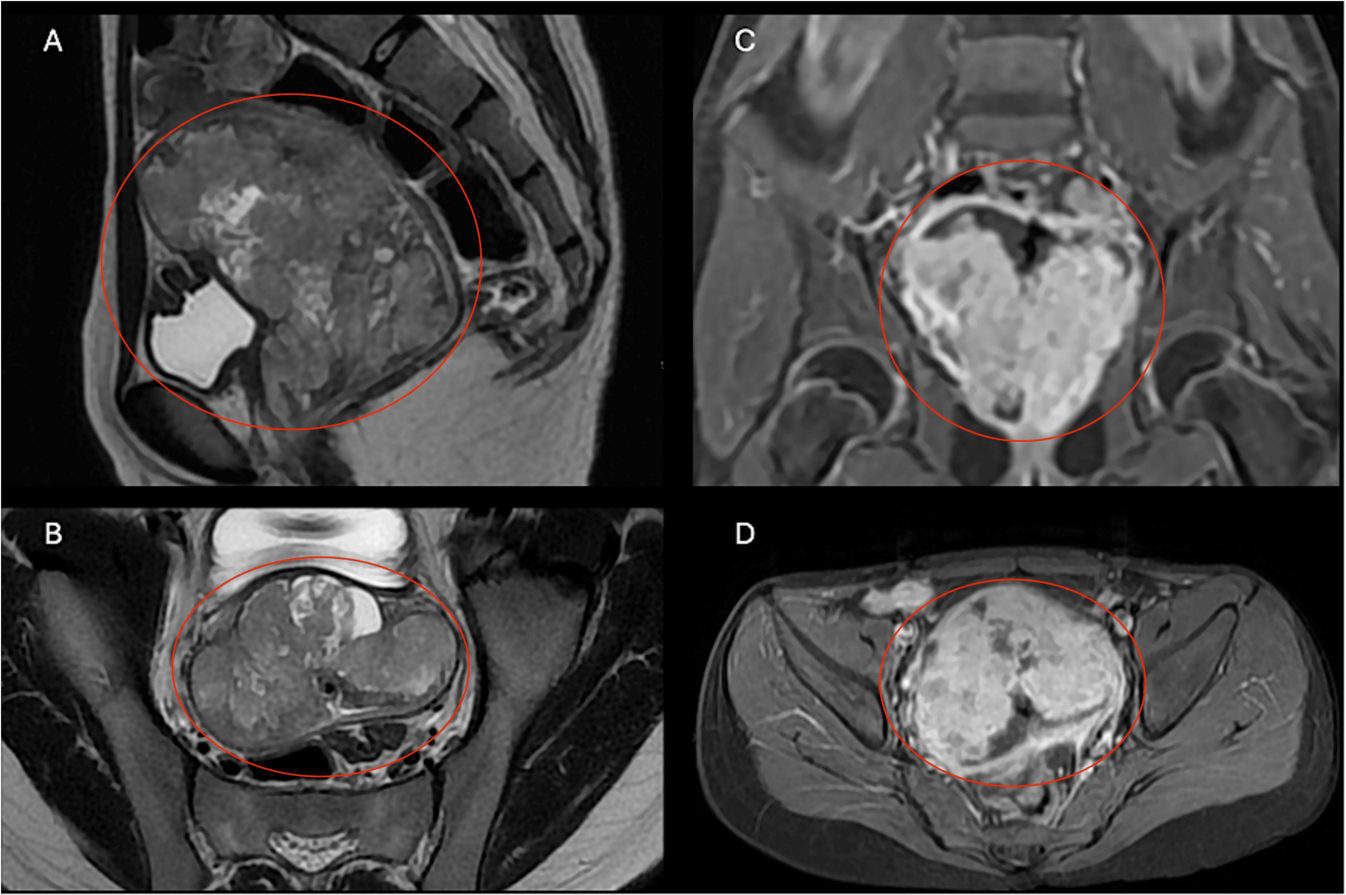
(A, B) T2-weighted sagittal and axial MRI showing a heterogeneous mass (red circles) occupying and distending the vaginal cavity without extension to adjacent structures; (C, D) T1-weighted fat-suppressed coronal and axial images demonstrating hypervascular enhancement of the vaginal lesion (red circles)

**Figure 2:**
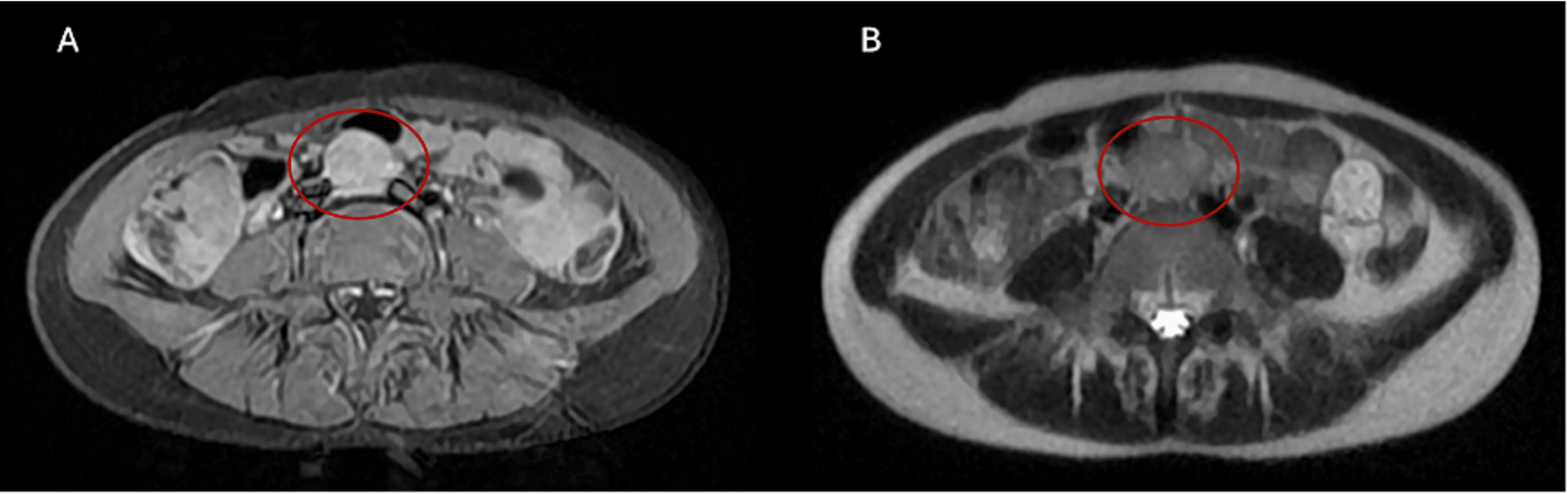
Axial T1 fat-suppressed postcontrast (A) and T2 (B) images showing a metastatic lymph node located below the aortic bifurcation (red circle)

A chest CT scan and abdominal/pelvic MRI confirmed a vaginal mass measuring approximately 80 × 49 × 82 mm with central necrosis and involvement of the vaginal capsule (Figure 1). A right para-aortic lymph node enlargement measuring 24 × 22 mm was also visible (Figure 2). The chest CT showed no evidence of metastatic lesions.

**Figure 3:**
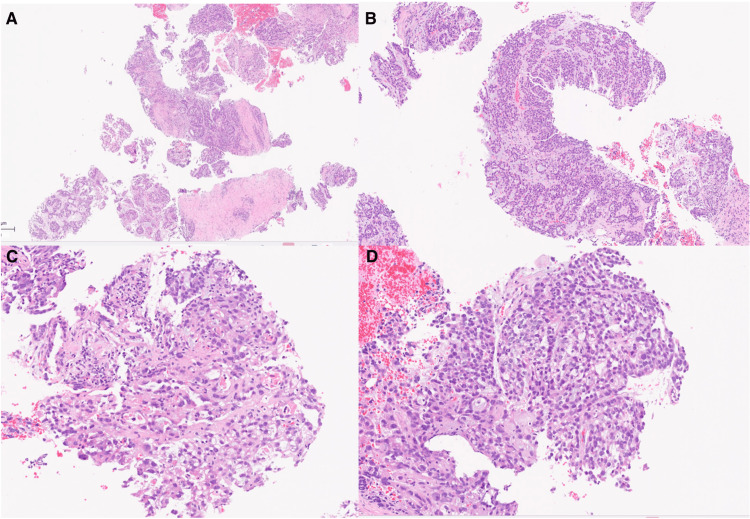
Histopathological features of the tumor (hematoxylin and eosin (HE) stain) (A) At 5X magnification, areas with tubulocystic, solid, and papillary histologic patterns are identified, associated with foci of necrosis; (B) At 10X magnification, areas showing tubulocystic and solid patterns of variable size are observed, with dense eosinophilic intraluminal protrusions lined by a single layer of cuboidal cells; (C) At 40X magnification, papillary areas with edematous stroma are observed, lined by one or two layers of cuboidal cells with clear to eosinophilic cytoplasm and uniformly atypical nuclei, consistent with a papillary pattern; (D) At 40X magnification, papillary areas with edematous stroma are observed, lined by one or two layers of cuboidal cells with clear to eosinophilic cytoplasm and uniformly atypical nuclei, consistent with a papillary and solid pattern.

**Figure 4:**
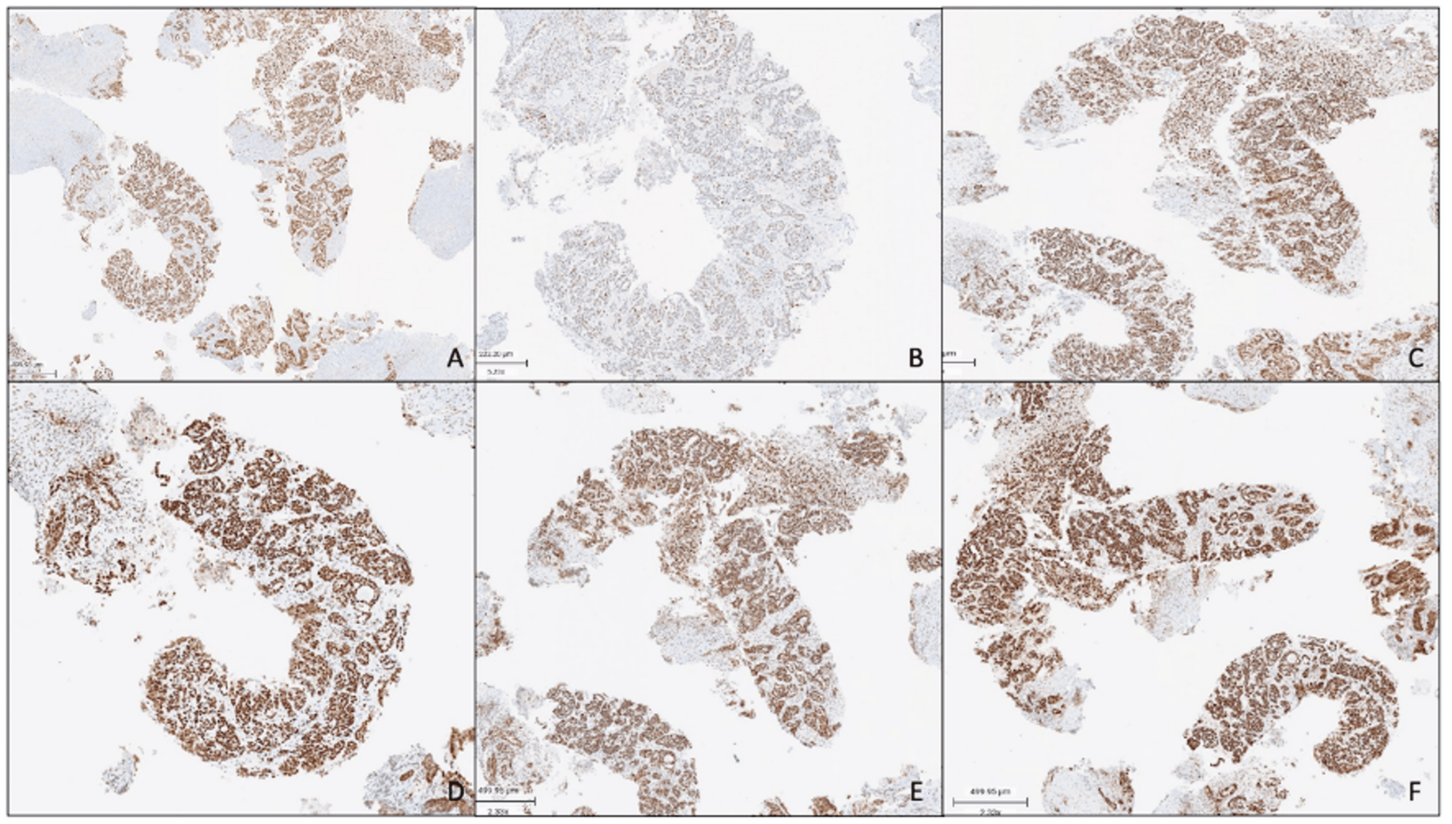
Immunohistochemistry studies (A) PAX8: Nuclear staining; (B) P53: No abnormal expression: wild-type pattern; (C) MSH2: Intact nuclear expression; (D) MSH6: Intact nuclear expression; (E) PMS2: Intact nuclear expression; (F) MLH1: Intact nuclear expression

The patient underwent an image-guided percutaneous biopsy without complications. Histopathological analysis confirmed clear cell carcinoma without aberrant p53 expression (Figure 3). Immunohistochemistry showed tumor cell positivity for CK7 and PAX8, focal mosaic positivity for p16, and preserved expression of DNA mismatch repair (MMR) proteins (Figure 4).

**Figure 5:**
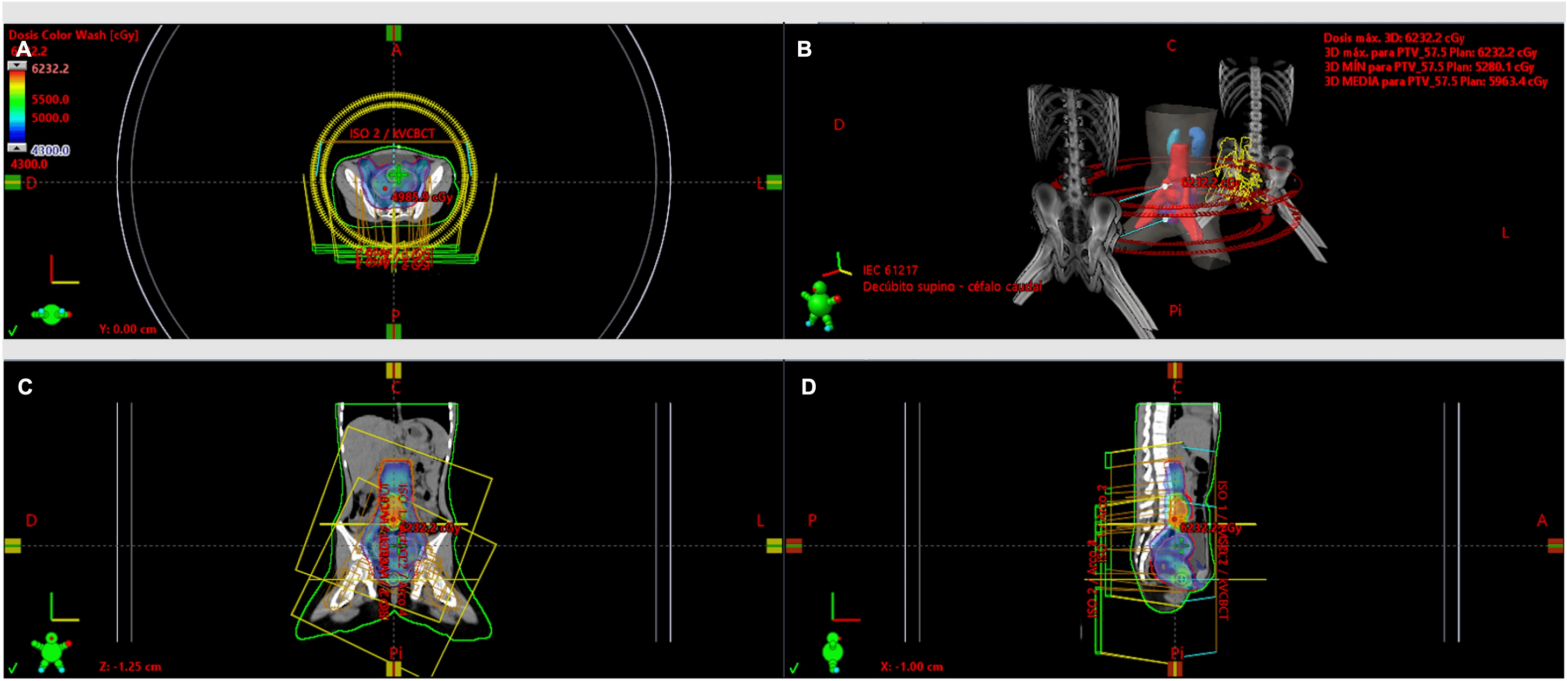
EBRT dosimetric plan using Eclipse™ (Varian Medical Systems, Palo Alto, CA, US) In the axial (A), coronal (C), and sagittal (D) views, 95% coverage of the prescribed dose over the planning target volume (PTV) is observed. A VMAT technique with four arcs (B) was used to optimize dose distribution and minimize toxicity to organs at risk. EBRT: external beam radiation therapy; VMAT: volumetric modulated arc therapy

A multidisciplinary tumor board, comprising pediatric oncology, pediatric surgery, radiation oncology, and gynecologic oncology, discussed the case. The disease was classified as FIGO (International Federation of Gynecology and Obstetrics) 2018 stage III and considered unresectable. The patient received concurrent chemoradiation with weekly cisplatin (40 mg/m²) for five weeks and image-guided external beam radiation therapy (EBRT) using volumetric modulated arc therapy (VMAT). She received a total dose of 45 Gy in 1.8 Gy daily fractions over the pelvic, para-aortic, and inguinal fields. Subsequently, a boost to 57.5 Gy (2.3 Gy/fraction) was administered to the involved common iliac lymph node chains, completing 25 sessions (Figure 5).

**Figure 6:**
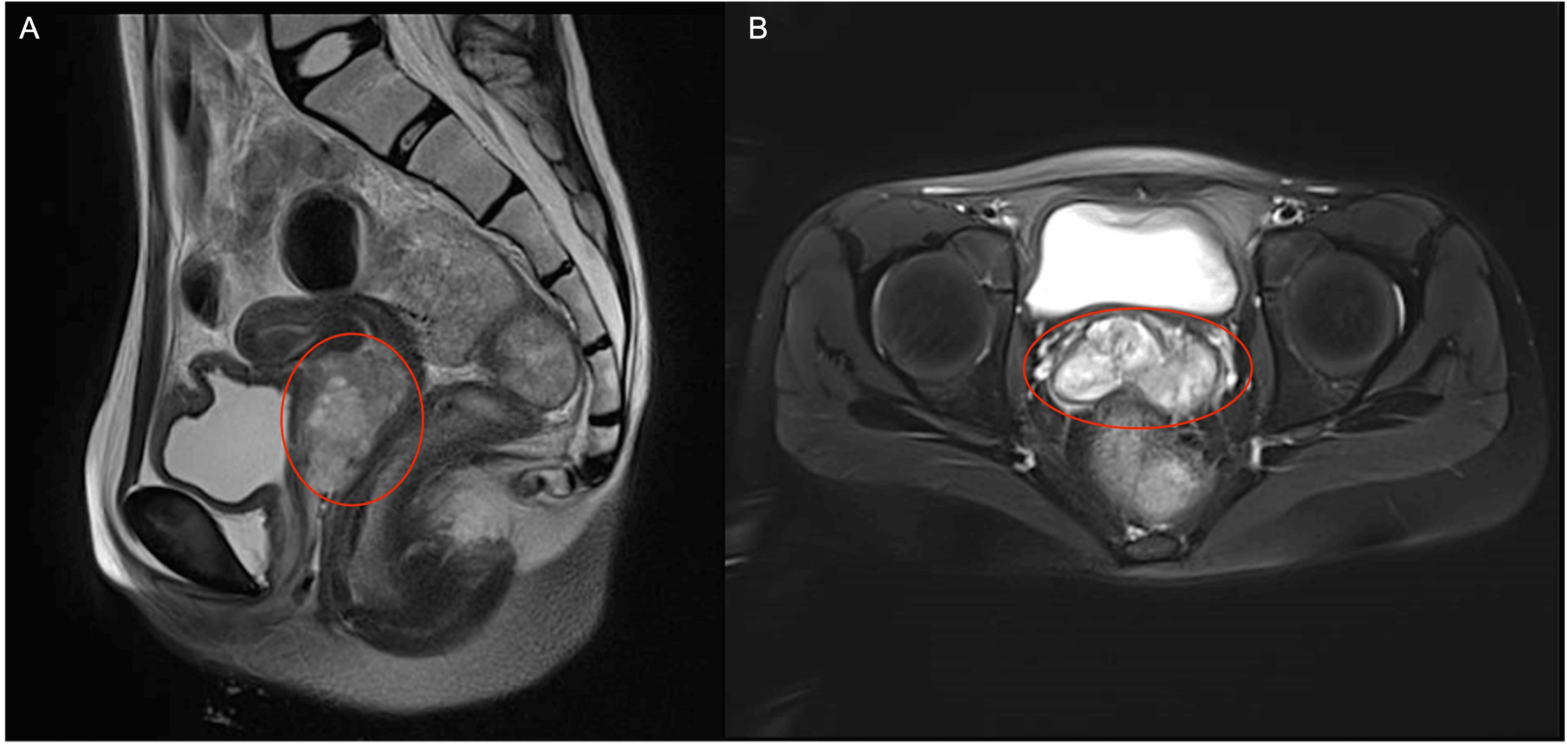
Contrast-enhanced pelvic MRI following cisplatin and EBRT showing the post-treatment residual lesion (red circles) (A) Sagittal view; (B) Axial view EBRT: external beam radiation therapy

At the end of EBRT, pelvic MRI demonstrated a significant reduction in tumor volume, but a residual lesion measuring 46 × 44 × 23 mm (approximately 47 cm³) persisted (Figure 6). Examination under sedation confirmed circumferential (360°) vaginal wall involvement. The patient then received high-dose-rate interstitial brachytherapy (HDR-ISBT) with Iridium-192, consisting of four applications of 6 Gy each.

The total duration from diagnosis to completion of treatment was approximately 13 weeks. The patient was diagnosed in June 2025, started chemotherapy later that month, completed external beam radiotherapy in August 2025, and finished interstitial brachytherapy in September 2025. Since then, and up to the time of this report, she remains under multidisciplinary follow-up with no clinical evidence of recurrence.

## Discussion

Clear cell carcinoma of the vagina occurs rarely in children, accounting for less than 10% of vaginal tumors, and generally exhibits aggressive behavior in this age group. It often appears as a large, polypoid, or nodular abdominal mass, though flat or ulcerated lesions have also been described [1]. Lymphatic and vascular spread may occur, with reported metastases to the lungs, kidneys, peritoneum, omentum, ovaries, liver, and brain [7]. Clinically, it manifests as abdominal pain, an abdominal or pelvic mass, bloody vaginal discharge, or a protruding vaginal lesion [2].

To the best of our knowledge, this is the first reported case of vaginal clear cell carcinoma in the pediatric population in Colombia. Historically, in utero exposure to DES has been linked with the development of this tumor, attributed to the persistence of Müllerian epithelium and abnormal epithelial-mesenchymal interaction in the vagina. However, CCC can occur even in the absence of DES exposure, arising from metanephric or mesonephric remnants or Müllerian duct abnormalities [8]. This theory may also explain the increased incidence of high-grade intraepithelial neoplasia (CIN2+) [9].

Recent evidence suggests that DES effects may extend beyond the directly exposed generation through epigenetic mechanisms such as alterations in DNA methylation. Nevertheless, an updated analysis of the National Cancer Institute (NCI) multigenerational cohort found no increased risk of CCC or other malignancies in third-generation descendants. However, long-term follow-up remains essential [10].

Magnetic resonance imaging is the imaging modality of choice for evaluating these lesions because of its superior soft-tissue contrast, allowing detailed assessment of the primary tumor, invasion into adjacent structures, and evaluation of pelvic and inguinal lymph nodes [6].

Cytologic and immunophenotypic features are crucial for differentiating CCC from rhabdomyosarcoma, germ cell tumors, and other clear cell adenocarcinomas. Among immunohistochemical markers, PAX8 is particularly important as a key indicator confirming Müllerian origin [8], as demonstrated in our case.

Regarding treatment, Bujor et al. reviewed the literature on adolescent cases, emphasizing the need for individualized management and noting that current evidence is mainly derived from case reports and small series [11]. Treatment planning typically relies on findings from adult populations, with adjustments made to fit each patient's unique clinical and histopathological profile.

In 2023, the European Society for Radiotherapy and Oncology (ESTRO), the European Society of Gynaecological Oncology (ESGO), and the European Society for Paediatric Oncology (SIOPe) published evidence-based guidelines for optimizing the management of vaginal cancer within a multidisciplinary framework [6]. Surgical management is advisable only for lesions ≤2 cm that do not invade adjacent organs. For cases with positive surgical margins or lymph node involvement, external beam radiation therapy (EBRT) with or without adjuvant chemotherapy is indicated. For tumors >2 cm and/or with nodal disease, EBRT with platinum-based chemotherapy followed by brachytherapy is the treatment of choice. In patients with oligometastatic disease, treatment may include chemoradiation and brachytherapy along with targeted metastasis-directed therapy. For disseminated metastatic disease, systemic therapy is the mainstay, along with palliative care for symptom control [6]. Following these recommendations, our patient received concurrent cisplatin-based EBRT followed by brachytherapy, which she tolerated well and to which she showed a favorable clinical response.

In adults, high-dose-rate interstitial brachytherapy (HDR-ISBT) is advisable for patients with gynecologic anatomy unsuitable for intracavitary implantation, bulky tumors (≥30 cm³), persistent parametrial involvement, or residual disease in the lower third of the vagina after EBRT. This technique ensures adequate tumor dose coverage while minimizing radiation exposure to adjacent organs [12,13]. Tumor size at diagnosis and before brachytherapy initiation is a significant predictor of local failure [14].

Ultimately, prognosis mainly depends on disease stage, tumor size, surgical margins, and nodal status. Localized cases that undergo complete surgical resection have favorable outcomes, whereas advanced disease is associated with lower survival rates [1,2,7].

## Conclusions

Raising awareness among healthcare professionals caring for pediatric and adolescent patients regarding the potential for vaginal malignancies is crucial. Diagnosis requires a high index of suspicion and confirmation by histologic and immunohistochemical studies. Management remains unstandardized: complete surgical excision with negative margins may be curative in early-stage disease, whereas combined chemoradiation is indicated in advanced cases.

Given the limited evidence and the absence of randomized studies for this rare pediatric tumor, our experience, together with existing reports, may contribute to the development of more specific treatment guidelines in the future.
